# Prevalence and Determinants of Chronic Kidney Disease among Patients with Sickle Cell Disease in Tanzania

**DOI:** 10.21203/rs.3.rs-8796287/v1

**Published:** 2026-03-27

**Authors:** Nyanjiga Mkangara, Florence Urio, Agness Jonathan, Emmanuella Ambrose, Lulu Chirande, Mwashungi Ally, Rittah Mutagonda, Emmanuella Marco, Benson Kidenya, Clara Chamba, Ahlam Nasser, Yonazi Mbonea, Emmanuel Balandya, Paschal Ruggajo

**Affiliations:** Muhimbili University of Health and Allied Sciences; Muhimbili University of Health and Allied Sciences; Sickle Pan-African Research Consortium; Sickle Pan-African Research Consortium; Muhimbili University of Health and Allied Sciences; Muhimbili University of Health and Allied Sciences; Muhimbili University of Health and Allied Sciences; Sickle Pan-African Research Consortium; Sickle Pan-African Research Consortium; Muhimbili University of Health and Allied Sciences; Muhimbili University of Health and Allied Sciences; Muhimbili National Hospital; Muhimbili University of Health and Allied Sciences; Sickle Pan-African Research Consortium

**Keywords:** Sickle cell disease, chronic kidney disease, microalbuminuria, urine creatinine, Tanzania

## Abstract

**Background:**

Chronic Kidney Disease (CKD) is a common complication in Sickle Cell Disease (SCD), accounting for 16–18% of attributable mortality among patients with SCD. Previous studies have identified microalbuminuria, diabetes mellitus (DM) and hypertension as early determinants of CKD in SCD, but few studies have been done in Tanzania to characterize the magnitude and risk factors for kidney disease in this patient population.

**Aim:**

This study aimed to assess the prevalence and determinants of kidney disease among SCD patients at Muhimbili National Hospital (MNH) in Dar es Salaam, Tanzania.

**Methods:**

We conducted a cross-sectional study involving 369 patients with SCD. Socio-demographic and clinical data including blood pressure and random blood glucose were collected. Blood and urine samples were analyzed for serum creatinine, urine creatinine, and urine albumin. Statistical analyses, including Chi-square tests and multivariable logistic regression, were performed using IBM SPSS v24.0 to explore the association between socio-demographic and clinicopathological parameters (age, sex, blood pressure, DM, microalbuminuria) with kidney disease (defined as presence of established structural damage of the kidneys or a decreased eGFR [< 60 mL/min/1.73m^2^] lasting for at least three months). A p-value < 0.05 was considered to be statistically significant.

**Results:**

The prevalence of kidney disease was 49%, with a higher prevalence (66.9%) among study participants aged between 5–18 years. Hypertension was present in 1.1%, DM in 0.5% and microalbuminuria in 30.6% of the study population. Patients with hypertension had a 4.2-fold (OR 4.22, CI 1.18–15.15) increased likelihood of having kidney disease whereas those with DM and microalbuminuria had 9.1-fold (OR 9.13, CI 2.62–31.9) and 2-fold (OR 2.67, CI 1.84–4.16) increased likelihood of having kidney disease, respectively. There was a 14.4% (p-<0.001) discrepancy in kidney disease detection based on urine creatinine compared to serum creatinine-based criteria.

**Conclusion:**

We report a high prevalence and early occurrence of kidney disease among patients with SCD in Tanzania. This study highlights the association between microalbuminuria and other clinical parameters such as hypertension and DM with the occurrence of kidney disease in patients with SCD, underscoring the need for early screening, evaluation and intervention for progressive chronic kidney disease among patients with SCD.

## Introduction

Sickle Cell Disease (SCD) is the most common cause of morbidity and mortality among monogenic disorders worldwide. SCD is disproportionately prevalent in Sub-Saharan Africa (SSA), where over 75% of the global patient population resides and where up to 3% of newborns are born with the disease in some areas(1). Estimates indicate that between 2010 and 2050, 14 million children will be born with homozygous SCD worldwide, with 84% of these cases likely to be from Africa(1). Tanzania has one of the highest annual birth rates for people with SCD, with an estimated 11,000 births per year(1). Homozygous SCD, defined by the presence of two copies of the globin S (S) mutation which encodes for sickle cell hemoglobin (Hb S), is the most frequent subtype of SCD globally. Children with SCD are at a high risk of morbidity and mortality, with the highest incidence of death reported in the first 5 years of life (2).

Kidney disease is prevalent among SCD patients, accounting for 16–18% of total mortality (3). The condition, also known as sickle cell nephropathy, is more common in homozygous SCD patients and can be less severe in compound heterozygous SCD (such as HbSC and HbS beta+ thalassemia) and comparatively milder in the heterozygous state (3). Kidney disease in individuals with SCD begins early in childhood, and symptoms include decreased urine concentrating ability, glomerular hyperfiltration and significantly increased albuminuria (3).

The mechanisms causing renal injury in SCD are not completely understood, although hemolysis, vaso-occlusion and ischemia are thought to play a role, resulting in a variety of functional abnormalities ranging from tubular and glomerular dysfunction to severe morphological changes in the kidneys (4). The hypoxic, acidic, and hyperosmolar environment in the inner medulla is known to promote red blood cell (RBC) sickling, resulting in impaired renal medullary blood flow, ischemia, microinfarction, and papillary necrosis (4). Hematuria is most usually caused by a vascular blockage with RBC extravasation into the collecting system or papillary necrosis. The altered pressure response to angiotensin II in SCD may also play a part in the pathogenesis of renal dysfunction (5).

To date, few studies have been conducted in SSA to ascertain the magnitude and the risk factors for kidney dysfunction among patients with SCD. This study aimed to provide insights on these critical determinants of kidney disease among patients with SCD in Tanzania with the goal of providing evidence for implementation of preventive and curative measures.

## Materials and methods

### Study design and study setting

This was a descriptive cross-sectional study conducted at Muhimbili National Hospital (MNH) in Dar-es-salaam, Tanzania. Patients were recruited at the SCD clinic at MNH which runs twice a week (on Tuesdays and Fridays) for adults and every Thursday for children. Follow-up visits are scheduled at an average of once every three months. A team of seven Specialist Hematologists as well as Hematology Residents attend about 30 adult and 40 pediatric SCD patients each week. The SCD clinic at MNH is integrated with the Sickle Pan-African Research Consortium (SPARCO)-Tanzania project which provides a platform for electronic recording of patients visits. Services provided during clinic visits include (but are not limited to) health education on SCD, anthropometric measurements, basic investigations such as full blood count, renal function tests and liver function tests, prescription of hydroxyurea and folic acid, as well as counseling on infection prevention. Patients in need of admission are transferred to inpatient care.

### Study population and sample size calculation

All patients (adults and children) with SCD who attended the clinic at MNH between from November 2022 and April 2023 were eligible for enrolment into the study. Sample size was calculated to be 369 patients.

### Data collection tool

A paper questionnaire was used to gather information on socio-demographic, clinical and laboratory parameters. The parameters included age, educational status, random blood sugar levels, serum creatinine, urine creatinine, glomerular filtration rate and blood pressure. Information was collected directly from study participants and from patients’ medical records.

### Blood collection and biochemical analysis

Two milliliters (2ml) of peripheral venous blood was drawn from each study participant and collected onto plain tube for isolation of serum and EDTA tubes for blood glucose testing. Analysis of serum creatinine was performed on the collected serum samples using the Chemistry Analyzer (Automated) (COBAS INTEGRA 400, Roche Diagnostics, Basel Switzerland). Blood samples in EDTA tubes were sent to the laboratory for random blood glucose testing.

### Urine collection and biochemical analysis

Each participant was asked to provide midstream urine sample using a clean urine container which was tested on-spot for presence of microalbumin and creatinine by using the URiSCAN Optima 7 point-of-care device (YD Diagnostics Co., Ltd, South Korea). Presence of glucose, ketones, macroalbuminuria, nitrites and red blood cells in urine samples was evaluated using conventional urine dipstick (Brain Solution).

### Ethical approval and patient consent

This study was approved by the MUHAS Institutional Review Board with the ethical clearance no MUHAS-REC-10–2022-1419. Written informed consent was obtained from the patients (above 18 years of age) as well as legally authorized representatives of children. To ensure privacy and confidentiality, consultations with study participants were conducted in a designated private office and respondents were identified by study numbers rather than names. Participants were assured that only aggregated and anonymized data will be used in research dissemination. The research was conducted observing the agreement of Helsinki declaration.

### Data Analysis

The collected data was transferred to SPSS version 24, then checked for completeness and consistency before analysis. For adults, the CKD-EPI formula was used to ascertain renal function via estimated Glomerular Filtration Rate (eGFR = 141 * min (Scr/,1) * max (Scr/κ, 1)^−1.209^ * 0.993^Age^ * 1.018 [if female] * 1.159 [if black])(6). For children, the modified pediatric Schwartz equation was used to calculate eGFR (eGFR = (k * height)/ Serum creatinine, where k = 0.45 for infants and 0.55 for those aged 1–12 years)(7). In this study, dependent variables were microalbuminuria and renal dysfunction, while independent variables were age, sex, blood pressure, DM defined as random blood sugar levels of 11.1mmol/L or higher. Renal dysfunction (or progressive kidney disease) was considered when urine albumin > 20 mg/l and/or urine albumin-to-creatinine ratio (ACR) > 30 mg/g. CKD stages were defined as follows: Stage 1 (eGFR ≥ 90), Stage 2 (eGFR 60–89), Stage 3a (eGFR 45–59), Stage 3b (eGFR 30–44), Stage 4 (eGFR 15–29) or Stage 5 (eGFR < 15).

The Chi-square test/Fisher exact test were used to determine the relationship between the ACR results and participant’s social demographic and clinical characteristics. A multivariable logistic regression model was fitted to identify predictors of kidney disease. Variables whose univariate analysis had a p-value < 0.25 were considered for multivariable analysis. However, other variables such as the use of medications such as hydroxyurea and level of education which did not meet the criteria were added to the model due to their clinical importance. Agreement (concordance) between urine creatinine and serum creatinine tests in detecting kidney disease was assessed via a proportion test. Differences were considered statistically significant when two-tailed *p*-values were < 0.05.

## Results

A total of 363 patients were recruited into the study. Majority of patients (66.9%) were between the ages of 5–18 years and 52.1% were females. More than half of the patients (59.2%) were on both folic acid and hydroxyurea (see Table 1).

### Prevalence of kidney disease among patients with SCD

The overall prevalence of kidney disease in the study population, via ACR, was 49.0%. The age group 5–18 years had the highest prevalence of 56.4% which was statistically different from the prevalence in the other age groups (p < 0.001) (Table 2).

### Determinants of kidney disease in patients with SCD

Table 3 presents the clinical and pathological markers associated with kidney disease. Patients at the age of 5–18 years had a 10-fold increased risk of developing kidney disease as compared to other age groups. Individuals with a secondary school education exhibited a 4.5-fold increased likelihood of having kidney disease compared to those with only primary education. Diabetic patients had a 9.1-fold higher likelihood of having kidney disease compared to non-diabetic individuals. Additionally, hypertensive individuals showed a 4.2-fold increased likelihood of having kidney disease compared to normotensive persons. Patients with microalbuminuria had a 2.6-fold higher likelihood of having kidney disease compared to those without microalbuminuria.

### Difference in prevalence between serum creatinine and urine creatinine

Table 4 we compared the prevalence of kidney disease between the two diagnostic tests: ACR and serum creatinine. The results showed that the prevalence of kidney disease, when diagnosed using serum creatinine, was significantly higher (PR = 63.4%, 95% CI: 58.4%–68.3%) compared to that obtained with URiSCAN (PR = 49.0%, 95% CI: 43.9%–54.2%). The difference in prevalence between the two methods was 14.4% (95% CI: 0.1%–0.2%). This indicates that relying on URiSCAN for creatinine measurement could result in missing approximately 14 cases of kidney disease out of 363 patients.(see Table 4)

## Discussion

Individuals with SCD are at a high risk for developing premature kidney dysfunction. In this study, we show that nearly half of individuals with SCD seen at MNH in Tanzania have kidney disease, which was more prevalent in younger patients aged 5–18 years. Further, we show that 1.1% of the patients had hypertension while 0.5% were diabetic and 30.6% had microalbuminuria, and these increased the odds of having kidney dysfunction 4.2-fold, 9.1-fold and 2-fold, respectively. There was a 14.4% discrepancy in kidney disease detection based on urine creatinine compared to serum creatinine-based criteria. These findings underscore the need for early screening, follow-up and interventions to prevent progressive kidney disease among patients with SCD.

Kidney disease is common among patients with SCD. Results from this study show that the prevalence of kidney disease was 49.0% compared to 31.4% that was reported by Kimaro *et al* in Mwanza, Tanzania in 2019 (4). Another study conducted earlier in 2021 by Saidia *et al* in Dar-es-salaam, Tanzania reported the prevalence of kidney disease of 14.7%. However, this study was limited by only including children below 10 years of age and used only eGFR and proteinuria to define kidney disease. Nonetheless, this study, alongside observation from our study that kidney disease was more prevalent in the 5–18 years of age, underscores earlier occurrence and need for early screening for kidney disease in our patient population. Overall, the prevalence of kidney disease reported in our patient population was generally higher than 12.3% reported in studies from the Democratic Republic of Congo (DRC) and 8.3% in a study done in USA. These differences reflect differences in methodology (cross sectional in current study compared to retrospective chart review in a study in DRC), also difference in the availability, accessibility and time at initiation of diagnostic and quality comprehensive SCD care such as newborn screening, hydroxyurea and chronic transfusion therapy which are largely lagging in most low resource settings such as Tanzania

In this study, the prevalence of kidney disease in patients who had microalbuminuria was 16.2%, compared to 26% reported by Niss O *et al* (8). In another study that was done by Ahmen *et al*, which involved 72 patients, 25% had microalbuminuria (10). This indicates the need and potential utility of introducing measures to monitor microalbuminuria as a simple and non-invasive way to monitor renal damage and renal dysfunction in SCD.

Kidney disease has become a serious public health problem. One way to reduce the economic burden of chronic kidney disease would be early screening, detection and intervention. And this can be made effective by knowing the factors that are associated with the development of this disease. In this study, we have shown that SCD patients with established hypertension had increased odds of developing kidney disease compared to their normotensive counterparts. This is similar to a study that was done by Rumezya *et al* which showed that smoking, obesity, hypertension, and DM were important risk factors for kidney disease.An uncontrolled diabetic and/or hypertensive patient can easily and quickly progress to end-stage kidney disease (13).

This study has shown that the prevalence of kidney diseases differs when diagnosed using urine createnine via UriScan devises compared to serum creatinine by 14.4%, implying that 14 patients with kidney disease may be missed when diagnosed using urine createnine. In a study by Ko *et al* which compared the diagnostic performance in routine urinalysis, similar devices performed well in comparison to conventional centralized laboratory analyzers when used in detecting erythrocytes and leukocytes. This shows the potential for use of these devices after validation of its sensitivity and specificity in larger studies. The use of these point-of-care tests will revolutionize early detection of kidney dysfunction in patients with SCD.

This study has a major strength in shedding light on markers and determinants of kidney disease in patients with SCD, and providing insights on the age-related risks across the entire age spectrum. Nonetheless, our study had a limitation of non-use of Oral Glucose Tolerance Test (OGTT) or fasting plasma glucose testing which may have led to missed cases of DM and an underestimation of its prevalence.

In conclusion, our study has reported that nearly half of SCD patients attended at MNH have kidney disease. The presence of hypertension, DM and microalbuminuria predicted the occurrence of renal dysfunction in this patient population. Our study also highlighted the need for further studies into the potential for use of point-of-care tests in detecting microalbuminuria which could have great utility as a non-invasive alternative for outpatient screening for renal dysfunction in patients with SCD.

## Figures and Tables

**Table 1:** Socio-demographic characteristics of study participants (n = 363)

Variable	Frequency	Percentage
**Sex**		
Male	174	47.9
Female	189	52.1
**Age group(years)**		
Less than 5	6	1.7
5–18	243	66.9
Above 18	114	31.4
**Level of education**		
Primary school	78	21.5
Secondary	219	60.3
University/college	10	2.8
No formal education	56	15.4
**Previous Renal diagnosis**		
Yes	3	0.8
No	360	99.2
**Medical history**		
Urinary tract infections	329	90.6
Family history	3	0.8
Blood in urine	1	0.3
None	30	8.3
**Current Medication**		
Folic acid	133	36.6
Folic acid and hydroxyurea	217	59.8
Not in medication	13	3.6
**Hypertensive**		
Yes	4	1.1
No	359	98.9
**Diabetic**		
Yes	2	0.5
No	361	99.5
**Presence of Microalbumin**		
No	252	69.4
Yes	111	30.6

**Table 2:** Prevalence of kidney disease among patients with sickle cell disease attending Muhimbili National Hospital (n = 363)

Variable	ACR results		*p*-value
	Normal n (%)	Abnormal n (%)	
Total	185(51.0)	178(49.0)	
**Sex**			
Male	86(49.4)	88(50.6)	0.574
Female	99(52.4)	90(47.6)	
**Age group**			
Less than 5	5(83.3)	1(16.7)	<0.001
5–18	106(43.6)	137(56.4)	
Above 18	74(64.9)	40(35.1)	
**Level of education**			
Primary school	55(70.5)	23(29.5)	<0.001
Secondary	95(43.4)	124(56.6)	
University/college	9(90.0)	1(10.0)	
Insufficient formal education	26(46.4)	30(53.6)	
**Renal diagnosis**			
Yes	3(100.0)	0(0.0)	0.248
No	182(50.6)	178(49.4)	
**Past medical history**			
Urinary tract infections	171(52.0)	158(48.0)	0.162
Family history	0(0.0)	3(100.0)	
Blood in urine	1(100.0)	0(0.0)	
None	13(43.3)	17(56.7)	
**Medications**			
Yes	179(51.0)	172(49.0)	0.946
No	6(50.0)	6(50.0)	
**Hypertension**			
Yes	3(75.0)	1(25.0)	0.623
No	181(50.6)	177(49.4)	
**Diabetes**			
Yes	1(50.0)	1(50.0)	0.742
No	183(50.8)	177(49.2)	
**Presence of microalbumin**			
No	92(36.4)	160(63.6)	<0.001
Yes	93(83.8)	18(16.2)	

**Table 3:** , Clinico-pathological markers of progressive kidney disease among patients with SCD (n = 363).

Variable	cOR(95% CI)	*p*-value	aOR(95% CI)	*p*-value
**Sex**				
Male	1.12(0.75–1.69)	0.574	0.81(0.48–1.37)	0.427
Female	1		1	
**Age group (years)**				
Less than 5	1		1	
5–18	6.46(0.74–56.1)	0.091	10.00(4.26–23.45)	<0.001
Above 18	2.70(0.30–23.9)	0.372	4.02(1.23–13.10)	0.021
**Level of education**				
Primary school	1		1	
Secondary	3.12(1.79–5.44)	<0.001	4.57(2.07–10.08)	<0.001
University/college	0.26(0.03–2.22)	0.221	0.59(0.18–1.88)	0.371
Insufficient formal education	2.76(1.35–5.65)	0.005	3.31(1.50–7.32)	0.003
Kidney disease diagnosis				
Yes	0.96(0.30–3.304)	0.946	1.33(0.33–5.42)	0.694
No			1	
**Family history anaemia**				
Yes	0.90(0.59–1.38)	0.637	0.79(0.48–1.31)	0.366
No	1		1	
**Hypertension**				
Yes	0.34(0.03–3.31)	0.353	4.22(1.18–15.15)	0.027
No	1		1	
**Diabetes**				
Yes	1.03(0.06–16.66)	0.981	9.13(2.62–31.90)	0.001
No	1		1	
**Presence of microalbumin**				
No	1		1	
Yes	1.90(1.16–3.18)	<0.001	2.67(1.84–4.16)	<0.001

**Table 4:** Prevalence of kidney disease detected via serum creatinine vs. urine creatinine

Variable	Prevalence	95% CI	*p*-value
S. Creatinine	63.4	58.4–68.3	
ACR	49.0	43.9–54.2	
**Difference**	14.4	0.1–0.2	<0.001

## Data Availability

The data used to support findings of this study is available from the corresponding author upon request
